# Alzheimer’s Disease Assessment Scale–Cognitive subscale variants in mild cognitive impairment and mild Alzheimer’s disease: change over time and the effect of enrichment strategies

**DOI:** 10.1186/s13195-016-0170-5

**Published:** 2016-02-12

**Authors:** Jana Podhorna, Tillmann Krahnke, Michael Shear, John E Harrison, for the Alzheimer’s Disease Neuroimaging Initiative

**Affiliations:** Boehringer Ingelheim Pharma GmbH & Co. KG, Binger Strasse 173, Ingelheim/Rhein, 55218 Germany; Cogitars GmbH, Heidelberg, Germany; Boehringer Ingelheim Pharma GmbH & Co. KG, Biberach/Riss, Germany; VU University Medical Center Amsterdam, Amsterdam, The Netherlands

**Keywords:** ADAS-Cog, Enrichment markers, Mild Alzheimer’s disease, Mild cognitive impairment, t-Tau/Aβ ratio, Decline over time

## Abstract

**Background:**

Development of new treatments for Alzheimer’s disease (AD) has broadened into early interventions in individuals with modest cognitive impairment and a slow decline. The 11-item version of the Alzheimer’s Disease Assessment Scale–Cognitive subscale (ADAS-Cog) was originally developed to measure cognition in patients with mild to moderate AD. Attempts to improve its properties for early AD by removing items prone to ceiling and/or by adding cognitive measures known to be impaired early have yielded a number of ADAS-Cog variants. Using Alzheimer’s Disease Neuroimaging Initiative data, we compared the performance of the 3-, 5-, 11- and 13-item ADAS-Cog variants in subjects with early AD. Given the interest in enrichment strategies, we also examined this aspect with a focus on cerebrospinal fluid (CSF) markers.

**Methods:**

Subjects with mild cognitive impairment (MCI) and mild AD with available ADAS-Cog 13 and CSF data were analysed. The decline over time was defined by change from baseline. Direct cross-comparison of the ADAS-Cog variants was performed using the signal-to-noise ratio (SNR), with higher values reflecting increased sensitivity to detect change over time.

**Results:**

The decline over time on any of the ADAS-Cog variants was minimal in subjects with MCI. Approximately half of subjects with MCI fulfilled enrichment criteria for positive AD pathology. The impact of enrichment was detectable but subtle in MCI. The annual decline in mild AD was more pronounced but still modest. More than 90 % of subjects with mild AD had positive AD pathology. SNRs were low in MCI but greater in mild AD. The numerically largest SNRs were seen for the ADAS-Cog 5 in MCI and for both the 5- and 13-item ADAS-Cog variants in mild AD, although associated confidence intervals were large.

**Conclusions:**

The possible value of ADAS-Cog expansion or reduction is less than compelling, particularly in MCI. In mild AD, adding items known to be impaired at early stages seems to provide more benefit than removing items on which subjects score close to ceiling.

**Electronic supplementary material:**

The online version of this article (doi:10.1186/s13195-016-0170-5) contains supplementary material, which is available to authorized users.

## Background

For the past two decades, the 11-item Alzheimer’s Disease Assessment Scale–Cognitive subscale (ADAS-Cog 11) has been a nearly ubiquitous measure of cognition in clinical trials of putative new therapies for Alzheimer’s disease (AD). However, recent use of this scale has revealed a number of limitations driven by a shift toward assessing and treating patients in earlier stages of the disease. One is the lack of sensitivity to detect change in early stages of AD, as it seems most sensitive when used in patients in the moderate stage of AD [i.e., Mini Mental State Examination (MMSE) score of 12–18] [1]. The second relates to measuring cognitive domains known to be impaired at the early stages, such as executive function [2–4], that are not captured by ADAS-Cog 11. As a result, subjects with mild cognitive impairment (MCI) tend to score at ceiling (i.e., score of 0 for ADAS-Cog) on eight of the 11 ADAS-Cog subtest items [5–7].

In response to criticism of the ADAS-Cog’s inability to measure relevant cognitive domains, Mohs et al. [8] suggested the use of additional tests, such as Digit Cancellation, Delayed Word Recall and a Maze test. This has led to the creation of ADAS-Cog variants such as the ADAS-Cog 13. A second approach to improving the scale’s sensitivity was to remove subtests prone to ceiling effects (i.e., ADAS-Cog 3). ADAS-Cog 3 tests solely memory, however. The ADAS-Cog 5 variant combines the ADAS-Cog 3 items with Delayed Recall and Digit Cancellation from ADAS-Cog 13. This tactic could improve detection of cognitive decline over time because it measures more relevant domains impacted in early stages of the disease than the ADAS-Cog 3. However, the sensitivities of these new variants to detect change over time have not been compared in patients with early AD.

In this study, we employed data from the Alzheimer’s Disease Neuroimaging Initiative (ADNI) database to address this issue, as described in the Methods section. Our aim was to evaluate which of the four variants (ADAS-Cog 3, ADAS-Cog 5, the original ADAS-Cog 11, and ADAS-Cog 13) best detects cognitive decline over time in subjects with MCI or mild AD. We hypothesised that the ‘tailored’ ADAS-Cog 5 variant would provide the best means of assessing decline in the subjects with early AD compared with the other variants. It should be noted that our goal was not to validate a new instrument. In addition, as enrichment should help to identify subjects with MCI with AD pathology [9–11], we evaluated whether it helps to improve the sensitivity of the ADAS-Cog variants to detect decline over time.

## Methods

### Data source^1^

*Data used in the preparation of this article were obtained from the Alzheimer’s Disease Neuroimaging Initiative (ADNI) database (adni.loni.usc.edu). The ADNI was launched in 2003 by the National Institute on Aging (NIA), the National Institute of Biomedical Imaging and Bioengineering (NIBIB), the Food and Drug Administration (FDA), private pharmaceutical companies and non-profit organizations, as a $60 million, 5-year public private partnership. The primary goal of ADNI has been to test whether serial magnetic resonance imaging (MRI), positron emission tomography (PET), other biological markers, and clinical and neuropsychological assessment can be combined to measure the progression of mild cognitive impairment (MCI) and early Alzheimer’s disease (AD). Determination of sensitive and specific markers of very early AD progression is intended to aid researchers and clinicians to develop new treatments and monitor their effectiveness, as well as lessen the time and cost of clinical trials.*

*The Principal Investigator of this initiative is Michael W. Weiner, MD, VA Medical Center and University of California–San Francisco. ADNI is the result of efforts of many coinvestigators from a broad range of academic institutions and private corporations, and subjects have been recruited from over 50 sites across the U.S. and Canada. The initial goal of ADNI was to recruit 800 subjects but ADNI has been followed by ADNI-GO and ADNI-2.*

*To date these three protocols have recruited over 1500 adults, ages 55 to 90, to participate in the research, consisting of cognitively normal older individuals, people with early or late MCI, and people with early AD. The follow up duration of each group is specified in the protocols for ADNI-1, ADNI-2 and ADNI-GO. Subjects originally recruited for ADNI-1 and ADNI-GO had the option to be followed in ADNI-2. For up-to-date information, see**www.adni-info.org**.*

### Trial registration details

The trial is registered at ClinicalTrials.gov under trial registration numbers NCT00106899 (date of registration 31 March 2005), NCT01231971 (date of registration 27 October 2010) and NCT01078636 (date of registration 1 March 2010).

Data from ADNI 1, ADNI Grand Opportunities (ADNI GO) and ADNI 2 were downloaded on 30 September 2014. Subjects with available ADAS-Cog 13 data (i.e., comprising ADAS-Cog 11 items plus Delayed Word Recall and Digit Cancellation) and with available baseline cerebrospinal fluid (CSF) biomarkers, both amyloid-β (Aβ) and total Tau (t-Tau), were included in the main analysis of subjects with mild AD or MCI.

Due to the variable amount of available data at later time points, our focus was on subjects with change from baseline to 12 months in mild AD and to 24 months in MCI. Data beyond 12 months for mild AD and beyond 24 months for MCI were considered for data description over time.

### Ethics, consent and permissions

The ADNI study was approved individually by the institutional review boards of all the participating institutions. Written informed consent was obtained from all participants at each site. Please see the Acknowledgements section for a list of institutional review boards that approved the study.

### Study participants

The general inclusion and exclusion criteria for the ADNI study and the screening procedures were described by Petersen et al. [12]. The original subject cohorts included age-matched cognitively healthy subjects, subjects with MCI and subjects with mild AD dementia. The subjects with MCI had MMSE scores of 24–30 and a Clinical Dementia Rating (CDR) global score of 0.5 with a mandatory requirement of the memory box score being 0.5 or greater. The subjects with MCI had to be largely intact with regard to functional performance and could not qualify for the diagnosis of dementia [13]. The subjects with mild AD had MMSE scores of 20–26, a CDR score 0.5 or 1 and met the National Institute of Neurological and Communicative Disorders and Stroke/Alzheimer’s Disease and Related Disorders Association criteria for probable AD [14]. Subjects with MCI and subjects with mild AD were also required to meet criteria for memory impairment on the Wechsler Memory Scale–Revised Logical Memory II subscale [12].

### Populations in the statistical assessment

The ‘MCI set’ comprised subjects with MCI with available ADAS-Cog 13 at baseline and 24 months as well as available baseline Aβ and t-Tau CSF biomarkers. The ‘AD set’ comprised subjects with mild AD with available ADAS-Cog 13 at baseline and 12 months as well as available baseline Aβ and t-Tau CSF biomarkers. The MCI and AD sets described above are referred to as ‘non-enriched’ because their clinical data were analysed regardless of their biomarker status. The ‘biomarker-positive sets’ (‘enriched’) comprised those subjects with a positive AD pathology as defined by the magnitude of selected baseline CSF biomarkers [15] or with the presence of a genetic risk marker apolipoprotein E ε4 (*ApoE4*):

- CSF t-Tau/Aβ ratio >0.39
- CSF Aβ_1–42_ < 192 pg/ml
- CSF phosphorylated Tau (p-Tau) >23 pg/ml
- CSF t-Tau >93 pg/ml
- ApoE4 risk (one or two copies of the *ApoE4* allele)

For the purposes of this article, a CSF t-Tau/Aβ ratio >0.39 was considered to be the main enrichment strategy because, in the context of MCI, this would fulfil the recent International Working Group 2 research criteria for prodromal AD [16]. These cohorts are labelled as MCI+ and AD+ in this publication.

The ‘biomarker-negative’ sets comprised the complementary groups that did not fulfil the baseline biomarker enrichment criteria for AD based on t-Tau/Aβ and thus were not classified as having prodromal AD. Only biomarker-negative subjects with MCI are presented (MCI−). There were very few biomarker-negative subjects with mild AD.

### Statistical methods

In this study, we compared the ADAS-Cog 3 [17], ADAS-Cog 13 [8], the traditional ADAS-Cog 11 [18, 19] and the ADAS-Cog 5 variants (Additional file 1) to see which of them had the best ability to demonstrate a change in subjects with mild AD or with MCI.

Values for ADAS-Cog total scores were calculated on the basis of individual items and were recorded as missing if at least one ADAS-Cog item was not available (affecting six subjects with MCI and seven with mild AD). CSF measurements were obtained from biomarker datasets, where the latest available recorded result of the baseline sample was selected in case of duplicates.

Change from baseline was calculated for each individual. The mean change from baseline was calculated by cohort for the MCI and mild AD sets, as well as for the individual enriched sets, to describe the decline over time.

Demographics were obtained at the baseline visit and described by their mean value and standard deviation for continuous variables or by percentage for categorical variables.

Sensitivity of the ADAS-Cog variants to show a change over time was assessed using the signal-to-noise ratio (SNR). The SNR is calculated as the estimated mean change from baseline divided by the corresponding standard deviation. A positive or negative number indicates the direction of change towards increase (worsening) or reduction (improvement) from the observed ADAS-Cog score at baseline. The SNR reflects changes in the outcome relative to its variability, thereby allowing for direct comparison of different ADAS-Cog variants with respect to their sensitivity to detect a change from baseline. An increased SNR, representing a higher sensitivity, might be expected after removal of items that affect the mean change from baseline minimally (e.g., consistently scoring at the ceiling) through an anticipated decrease in variability that should be associated with a total score containing fewer individual items.

The mean change in ADAS-Cog score and the standard deviation were estimated from an analysis of covariance model correcting for baseline ADAS-Cog and MMSE scores, age, sex and *ApoE4* risk category. Variability of SNR estimates was captured using 95 % bootstrap confidence intervals based on 1000 samples for each set.

The four different ADAS-Cog variants were compared using a hypothetical patient, which was an *ApoE4*-positive 75-year-old woman with baseline MMSE and ADAS-Cog scores in line with the diagnostic group. Specifically, for subjects with MCI, the estimated baseline MMSE of 28 and ADAS-Cog 11 score of 10 were used. For subjects with mild AD, a baseline MMSE of 23 and ADAS-Cog 11 score of 18 were assumed. The baseline ADAS-Cog score for the remaining ADAS-Cog variants was estimated from a linear regression of baseline values of the respective ADAS-Cog variant vs. baseline ADAS-Cog 11.

Differences in SNR between pairs of ADAS-Cog variants were assessed by means of *p* values obtained from a paired *t* test accounting for correlated data, making use of covariance estimates obtained from the aforementioned bootstrapping samples. The reported *p* values are not corrected for multiplicity and are considered exploratory.

The analyses described above were performed on ADAS-Cog variants for the group of enriched subjects and separately for the group of non-enriched subjects.

No imputations for missing data or for discontinued subjects were performed; subjects with missing information were excluded from the analysis.

## Results

### Datasets analysed

There were 634 subjects with MCI for whom all baseline and month 24 ADAS-Cog 13 values were available. Of these, 382 (60 %) provided a CSF sample at baseline. Of the 242 subjects with mild AD for whom all baseline and month 12 ADAS-Cog 13 values were available, 97 (40 %) provided a CSF sample at baseline. The 229 subjects with MCI and 73 with mild AD who did not have ADAS-Cog 13 values at 24 or 12 months, respectively, were not included in the analyses. The number of subjects attending follow-up visits diminished over time. To maintain a sufficient number of subjects in the analyses, the focus was on change from baseline to 12 months in mild AD, while the change to 24 months was analysed for subjects diagnosed with MCI.

### Baseline characteristics

The baseline characteristics in the MCI and mild AD cohorts were similar, except that subjects with mild AD were older and more cognitively impaired (Table 1).

**Table 1:** Demographic and clinical data at baseline

	MCI	MCI+	MCI−	Mild AD	Mild AD+
Number of subjects (100 %)	382	206	176	97	90
Female (%)	43 %	42 %	45 %	45 %	48 %
Caucasian, *n* (%)	94 %	97 %	90 %	96 %	96 %
Age, yr	71.97 ± 7.39	72.97 ± 7.10	70.80 ± 7.56	75.17 ± 7.70	74.85 ± 7.66
Education, yr	16.28 ± 2.59	16.31 ± 2.62	16.25 ± 2.55	15.52 ± 2.60	15.51 ± 2.58
ApoE4 at risk,^a^ *n* (%)	177 (46 %)	142 (69 %)	35 (20 %)	67 (69 %)	65 (72 %)
MMSE	27.85 ± 1.75	27.41 ± 1.82	28.37 ± 1.52	23.19 ± 1.99	23.14 ± 1.99
CDR global	0.50 ± 0.00	0.50 ± 0.00	0.50 ± 0.00	0.77 ± 0.28	0.77 ± 0.28
CDR-SB	1.40 ± 0.84	1.56 ± 0.92	1.21 ± 0.70	4.40 ± 1.71	4.41 ± 1.65
ADAS-Cog 11	9.50 ± 4.29	10.83 ± 4.46	7.94 ± 3.50	19.66 ± 6.30	20.14 ± 6.26
ADAS-Cog 3	8.23 ± 3.76	9.43 ± 3.92	6.82 ± 3.02	15.95 ± 4.15	16.28 ± 4.12
ADAS-Cog 5	13.96 ± 6.17	16.12 ± 6.28	11.43 ± 4.99	26.20 ± 5.31	26.62 ± 5.23
ADAS-Cog 13	15.23 ± 6.68	17.52 ± 6.81	12.55 ± 5.43	29.91 ± 7.44	30.52 ± 7.35

*Aβ* amyloid-β, *AD* Alzheimer’s disease, *ADAS-Cog* Alzheimer’s Disease Assessment Scale–Cognitive subscale, *ApoE4*, apolipoprotein E ε4, *CDR* Clinical Dementia Rating, *MCI* mild cognitive impairment, *MMSE* Mini Mental State Examination, SB Sum of Boxes

Populations enriched based on t- Tau/Aβ ratio are labelled as MCI+ and AD+. Data are presented as mean ± standard deviation unless indicated otherwise

^a^ApoE4 status is not available for one subject in the MCI group

The 206 MCI+ (t-Tau/Aβ >0.39) set had baseline demographic characteristics similar to those of the non-enriched set, with the exception that 69 % of MCI+ subjects were *ApoE4*-positive vs. only 20 % of MCI− subjects. The baseline MMSE and CDR scores were also similar between the groups. The MCI+ set, however, was more impaired on those ADAS-Cog variants which include the additional measures of Delayed Word Recall and Digit Cancellation (e.g., ADAS-Cog 13 and the ‘tailored’ ADAS-Cog 5). The data in mild AD and mild AD+ sets are almost identical, as the majority of subjects with mild AD enrolled in ADNI studies had positive AD pathology at baseline (>90 %).

### Performance of ADAS-Cog variants in MCI and mild AD populations with and without enrichment

As expected, the magnitude of the absolute change on ADAS-Cog in mild AD is larger than that in MCI. As shown in Table 2, there was practically no decline in mean ADAS-Cog 11 score in subjects with MCI (worsening of 0.9 on a 70-point scale over 24 months and 1.9 points over 36 months). The change from baseline was modest in patients with mild AD (worsening of 3.5 points over 12 months and of 8.3 points over 24 months) (Additional file 2). This was also reflected on the MMSE (Additional file 3).

**Table 2:** ADAS-Cog 11 scores at each visit and change from baseline for MCI patients

	MCI (non-enriched)			MCI+ (enriched)			MCI− (‘biomarker-negative’)		
	Number of subjects	ADAS-Cog 11	CFB	Number of subjects	ADAS-Cog 11	CFB	Number of subjects	ADAS-Cog 11	CFB
Baseline/screen	382	9.5 ± 4.29		206	10.83 ± 4.46		176	7.94 ± 3.49	
Month 6	376	9.6 ± 4.75	0.0 ± 3.40	204	11.16 ± 4.82	0.3 ± 3.61	172	7.70 ± 3.91	−0.31 ± 3.10
Month 12	380	9.3 ± 5.23	−0.2 ± 3.68	206	11.08 ± 5.54	0.3 ± 4.06	174	7.27 ± 3.95	−0.68 ± 3.10
Month 24	382	10.4 ± 6.40	0.9 ± 4.45	206	12.74 ± 6.87	1.9 ± 4.92	176	7.70 ± 4.48	−0.24 ± 3.51
Month 36	169	10.8 ± 7.06	1.9 ± 5.45	89	13.83 ± 7.86	3.7 ± 6.21	80	7.40 ± 3.90	−0.26 ± 3.41

*ADAS-Cog* Alzheimer’s Disease Assessment Scale–Cognitive subscale, *CFB* change from baseline, *MCI* mild cognitive impairment

Increased score on ADAS-Cog 11 (maximum total score 70) indicates cognitive worsening. Data are presented as mean ± SD

On ADAS-Cog 11, enrichment minimally increased the decline in subjects with MCI over 24 months (Table 2). Even after 36 months in MCI+ subjects, the decline on ADAS-Cog 11 was less than 4 points. As shown in Table 3, there was a minimal decline over 24 months and a minimal impact of enrichment on the other ADAS-Cog variants (3-, 5- and 13-item) in subjects with MCI. As expected, there was no change in any of the ADAS-Cog variants in the subjects with MCI without AD pathology (MCI−) up to 36 months.

**Table 3:** ADAS-Cog scores and change from baseline up to 36 months for patients with MCI

	ADAS-Cog 3			ADAS-Cog 5			ADAS-Cog 13		
	Number of subjects	Score	CFB	Number of subjects	Score	CFB	Number of subjects	Score	CFB
MCI (non-enriched)									
Baseline/screen	382	8.23 ± 3.76		382	13.96 ± 6.17		382	15.23 ± 6.68	
Month 6	376	8.30 ± 4.14	0.01 ± 2.99	375	14.00 ± 6.68	−0.07 ± 4.01	375	15.28 ± 7.25	−0.07 ± 4.37
Month 12	381	8.09 ± 4.42	−0.15 ± 3.09	380	13.72 ± 7.09	−0.23 ± 3.95	379	14.97 ± 7.83	−0.24 ± 4.49
Month 24	382	8.94 ± 5.23	0.71 ± 3.56	382	15.09 ± 8.30	1.13 ± 4.87	382	16.57 ± 9.41	1.34 ± 5.68
Month 36	169	9.02 ± 5.34	1.23 ± 4.00	168	15.11 ± 8.58	1.95 ± 5.58	168	16.89 ± 10.18	2.59 ± 6.89
MCI+ (enriched)									
Baseline/screen	206	9.43 ± 3.92		206	16.12 ± 6.28		206	17.52 ± 6.81	
Month 6	204	9.71 ± 4.28	0.23 ± 3.17	203	16.38 ± 6.75	0.14 ± 4.18	203	17.84 ± 7.22	0.19 ± 4.56
Month 12	206	9.57 ± 4.63	0.14 ± 3.43	205	16.24 ± 7.21	0.16 ± 4.10	205	17.75 ± 8.01	0.28 ± 4.69
Month 24	206	10.91 ± 5.42	1.48 ± 3.78	206	18.32 ± 8.49	2.21 ± 5.20	206	20.15 ± 9.85	2.63 ± 6.24
Month 36	89	11.37 ± 5.72	2.55 ± 4.40	89	18.96 ± 9.00	3.82 ± 6.03	89	21.42 ± 11.01	5.02 ± 7.60
MCI− (biomarker − negative)									
Baseline/screen	176	6.82 ± 3.02		176	17.52 ± 6.81		176	12.55 ± 5.43	
Month 6	172	6.62 ± 3.25	−0.24 ± 2.75	172	17.84 ± 7.22	−0.31 ± 3.79	172	12.26 ± 6.03	−0.38 ± 4.12
Month 12	175	6.35 ± 3.45	−0.50 ± 2.69	175	17.75 ± 8.01	−0.68 ± 3.73	174	11.69 ± 6.20	−0.85 ± 4.18
Month 24	176	6.63 ± 3.88	−0.19 ± 3.06	176	20.15 ± 9.85	−0.11 ± 4.12	176	12.37 ± 6.80	−0.18 ± 4.51
Month 36	80	6.40 ± 3.35	−0.25 ± 2.88	79	21.42 ± 11.01	−0.16 ± 4.15	79	11.78 ± 5.95	−0.15 ± 4.69

*ADAS-Cog* Alzheimer’s Disease Assessment Scale–Cognitive subscale, *CFB* change from baseline, *MCI* mild cognitive impairment

Data are presented as mean ± standard deviation. Increased score on ADAS-Cog indicates cognitive worsening

Changes over time on other ADAS-Cog variants were modest (4.35 points over 12 months on ADAS-Cog 13) in subjects with mild AD (Table 4). Even though the number of subjects with mild AD attending a visit at 24 months was low, there was a trend toward a larger decline over 24 months with almost a 10-point decline on ADAS-Cog 13.

**Table 4:** ADAS-Cog score and change from baseline up to 24 months for subjects with mild AD

	ADAS-Cog 3			ADAS-Cog 5			ADAS-Cog 13		
	Number of subjects	Score	CFB	Number of subjects	Score	CFB	Number of subjects	Score	CFB
Mild AD (non-enriched)									
Baseline/screen	97	15.95 ± 4.15		97	26.20 ± 5.31		97	29.91 ± 7.44	
Month 6	95	16.95 ± 4.83	1.10 ± 3.85	95	27.62 ± 6.25	1.52 ± 4.32	95	31.86 ± 8.51	2.10 ± 4.79
Month 12	97	17.77 ± 5.29	1.82 ± 3.91	97	28.84 ± 6.75	2.64 ± 4.39	97	34.26 ± 10.43	4.35 ± 5.89
Month 24	40	18.68 ± 5.82	3.81 ± 5.12	40	30.27 ± 7.20	5.48 ± 6.13	38	37.17 ± 12.38	9.46 ± 9.15
Mild AD+ (enriched)									
Baseline/screen	90	16.24 ± 4.12		90	26.62 ± 5.23		90	30.52 ± 7.35	
Month 6	88	17.17 ± 4.89	1.03 ± 3.92	88	27.92 ± 6.32	1.41 ± 4.37	88	32.36 ± 8.58	1.99 ± 4.84
Month 12	90	18.19 ± 5.17	1.96 ± 3.98	90	29.42 ± 6.59	2.80 ± 4.46	90	35.08 ± 10.34	4.57 ± 6.03
Month 24	37	19.15 ± 5.77	4.06 ± 5.23	35	30.79 ± 7.18	5.63 ± 6.34	35	37.99 ± 12.51	9.77 ± 9.40

*AD* Alzheimer’s disease, *ADAS-Cog* Alzheimer’s Disease Assessment Scale–Cognitive subscale, *CFB* change from baseline

Data are presented as mean ± standard deviation. Increased score on ADAS-Cog indicates cognitive worsening

**Fig. 1:**
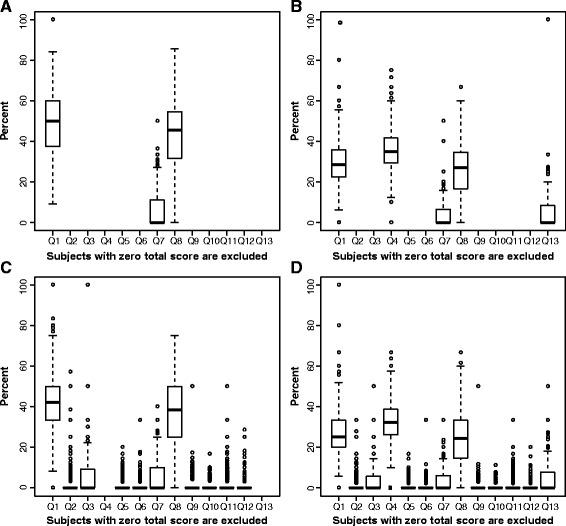
Relative percentage contribution of individual items to total Alzheimer’s Disease Assessment Scale–Cognitive subscale (ADAS-Cog) score at baseline for mild cognitive impairment population. **a** ADAS-Cog 3. **b** ADAS-Cog 5. **c**. ADAS-Cog 11. **d** ADAS-Cog 13

As the majority of subjects with mild AD had positive AD pathology at baseline (>90 %), no benefit of enrichment could be detected with respect to the increased magnitude of change over time (Additional file 2). Analysis of the contribution of individual items showed that Word Recall (Q1), Delayed Word Recall (Q4) and Word Recognition (Q8) largely contributed to the overall ADAS-Cog score (Fig. 1). Delayed Word Recall was impaired early and contributed largely to the overall score of the ADAS-Cog variants containing this item (i.e., the 13- and 5-item variants). Thus, somewhat counterintuitively, the score of the ADAS-Cog 5 exceeded that of the ADAS-Cog 11, presumably due to the contribution of Delayed Word Recall.

### Comparison of ADAS-Cog variants in MCI and mild AD populations with and without enrichment using SNR

To account for differences in possible maximum range and the variability in change from baseline, and hence to allow for cross-comparison of the various ADAS-Cog variants, SNRs were estimated on the basis of an analysis of covariance approach, correcting for baseline age, baseline MMSE, sex and *ApoE4* risk status. Specific comparisons of estimated SNRs across ADAS-Cog variants were made for a hypothetical patient as described in the Methods section. Table 5 shows the estimated SNRs for subjects with MCI, jointly presented with the corresponding bootstrap 95 % confidence intervals. Similarly, Table 6 shows results for subjects diagnosed with mild AD.

**Table 5:** Signal-to-noise ratio for change in ADAS-Cog variants over 24 months in MCI cohorts

	ADAS-Cog 3		ADAS-Cog 5		ADAS-Cog 11		ADAS-Cog 13	
	SNR	95 % CI	SNR	95 % CI	SNR	95 % CI	SNR	95 % CI
MCI	0.42	0.20–0.61	0.42	0.19–0.63	0.37	0.15–0.57	0.39	0.16–0.60
t-Tau/Aβ	0.43	0.15–0.69	0.45	0.16–0.73	0.39	0.11–0.65	0.42	0.12–0.69
Aβ	0.47	0.22–0.70	0.47	0.21–0.70	0.41	0.17–0.64	0.44	0.19–0.67
ApoE4+	0.53	0.24–0.82	0.61	0.33–0.88	0.53	0.26–0.78	0.60	0.33–0.86
t-Tau	0.45	0.14–0.75	0.52	0.18–0.84	0.39	0.09–0.66	0.45	0.14–0.77
p-Tau	0.43	0.19–0.65	0.43	0.17–0.66	0.37	0.12–0.61	0.39	0.14–0.63

*Aβ* amyloid β, *ADAS-Cog* Alzheimer’s Disease Assessment Scale–Cognitive subscale, *ApoE4* apolipoprotein E ε4, *CI* confidence interval, *MCI* mild cognitive impairment, *MMSE* Mini Mental State Examination, *SNR* signal-to-noise ratio

SNR estimates corrected for covariates, reported for an ApoE4-positive 75-year-old woman, baseline ADAS-Cog 11 of 10, baseline MMSE of 28. CIs were obtained via bootstrapping

**Table 6:** Signal-to-noise ratio for change in ADAS-Cog variants over 12 months in mild AD cohorts

	ADAS-Cog 3		ADAS-Cog 5		ADAS-Cog 11		ADAS-Cog 13	
	SNR	95 % CI	SNR	95 % CI	SNR	95 % CI	SNR	95 % CI
Mild AD	0.81	0.43–1.09	0.93	0.52–1.22	0.87	0.46–1.13	0.98	0.58–1.26
t-Tau/Aβ	0.83	0.41–1.10	0.95	0.53–1.23	0.86	0.46–1.12	0.96	0.55–1.24
Aβ	0.86	0.45–1.14	0.98	0.52–1.27	0.88	0.46–1.14	0.99	0.56–1.26
ApoE+	0.87	0.37–1.20	1.01	0.50–1.35	1.05	0.56–1.36	1.16	0.66–1.50
t-Tau	0.89	0.38–1.21	1.13	0.55–1.46	0.86	0.39–1.16	1.04	0.49–1.35
p-Tau	0.82	0.42–1.10	0.96	0.50–1.24	0.87	0.46–1.16	0.99	0.58–1.27

*Aβ* amyloid β; *AD* Alzheimer’s disease, *ADAS-Cog* Alzheimer’s Disease Assessment Scale–Cognitive subscale, *CI* confidence interval, *MMSE* Mini Mental State Examination, *SNR* signal-to-noise ratio

SNR estimates corrected for covariates, reported for an ApoE4-positive 75-year-old woman, baseline score corresponding to ADAS-Cog 11 of 18, baseline MMSE score of 23. CIs were obtained via bootstrapping

**Fig. 2:**
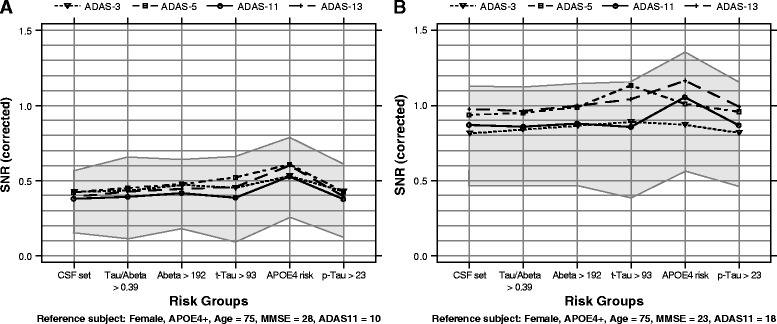
Signal-to-noise ratios (SNRs) for reference patients with mild cognitive impairment (MCI) and subjects with mild Alzheimer’s disease (AD). SNRs were corrected for change from baseline (CFB) for reference patients with **a** MCI at 24 months and **b** mild AD at 12 months. *Shaded area* represents bootstrap 95 % confidence intervals (CIs) of SNR for 11-item Alzheimer’s Disease Assessment Scale–Cognitive subscale (ADAS-Cog11) for each enrichment group. Bounds of CIs are connected solely for display purposes. *Abeta* amyloid-β, *ApoE4* apolipoprotein E ε4, *CSF* cerebrospinal fluid, *p-Tau* phosphorylated Tau, *t-Tau* total Tau, *MMSE* Mini Mental State Examination

Higher SNR values reflect increased sensitivity to detect a change. In subjects with MCI, SNRs based on estimated change from baseline to 24 months ranged between 0.37 and 0.61 (Table 5). The numerically largest SNRs were seen for the enriched set with *ApoE4* across all variants, and when we compared across subject sets, for the ADAS-Cog 5. However, the 95 % confidence intervals were broad (e.g., 0.33–0.88 for ADAS-Cog 5 on *ApoE4+* set) and almost identical to ADAS-Cog 13 and thus did not suggest that SNRs differed substantially among the variants. Overall, estimated SNRs were relatively similar, when looking at both different variants and different enrichment strategies (Fig. 2). A more formal comparison based on paired *t* tests was done (Additional file 4).

For subjects with mild AD at the 12-month time point, the estimated SNR values ranged from 0.81 to 1.16 (Table 6). Similarly to MCI, the numerically highest SNRs were seen for the *ApoE4*-enriched set across all variants. Across all subject sets, the highest SNRs were seen for both the 5-item and 13-item variants. The highest SNR (1.16) was observed in the *ApoE4*-enriched set on ADAS-Cog 13. The 95 % confidence intervals were also broad and overlapping in mild AD.

SNRs are graphically displayed in Fig. 2. SNRs in mild AD (Fig. 2, *right panel*) were larger than in subjects with MCI (Fig. 2, *left panel*), indicating that all ADAS-Cog variants are more sensitive to detect changes in subjects with mild AD than in subjects with MCI. SNRs of the variants fall within the 95 % bootstrap confidence intervals of the ADAS-Cog 11 (Fig. 2, *shaded area*).

A more formal comparison of pairs of variants was done based on *t* statistics for the difference in SNRs. Resulting *p* values (corrected not for multiplicity, but for correlation between variants) are reported in Additional file 4: Table S4. As usual, small *p* values indicate a higher chance of a true difference between the particular variants.

Among the various MCI subsets, there is no evidence indicating a difference in SNR between any of the ADAS-Cog variants based on the available ADNI data. All *p* values were larger than 0.10 for comparisons to the original ADAS-Cog 11.

In mild AD, the strongest evidence for a difference based on *p* values (<0.10) was found when we compared the 13- vs. 11-item and the 5- vs. 3-item variants for the t-Tau- and p-Tau-enriched sets (Additional file 4). This indicates that the difference between variants may be driven by the two additional items (Delayed Word Recall and Digit Cancellation) that are not included in the 3- and 11-item variants. When the accepted enrichment strategy of t-Tau/Aβ ratio was used, the *p* values were above 0.10.

## Discussion

ADAS-Cog has been the standard measure of cognition in AD clinical trials [20], but recently conducted trials with therapies aimed at slowing down disease progression in subjects at early stages of the disease revealed the limited sensitivity of the original ADAS-Cog 11 to detect change. Sensitivity to change may be compromised by the lack of tests to assess cognitive domains known to be impaired early in the disease, such as attention and executive functions [4], which has been addressed by adding tests to the original instrument [8] (e.g., the ADAS-Cog 13). A second approach to improve sensitivity of the instrument for early AD was to remove items prone to ceiling effects (e.g., ADAS-Cog 3). Data from subjects that score at ceiling inflate variance with negligible benefits in measuring change over time or treatment difference, particularly the possibility to detect improvement in cognition. The ADAS-Cog 3 extracted the items focused on memory (Word Recall, Orientation and Word Recognition) that are among the earliest manifestations of AD [4] and detect impairment in subjects with MCI approximately midway between normal healthy and patients with mild AD [6]. However, this approach is focused solely on a memory measure without considering other important areas of cognition affected at early stages of disease and therefore may not be optimal. The ADAS-Cog 5 combines both approaches and could theoretically be more sensitive than other variants to detect a change over time. It should be noted that our goal was not to validate a new instrument.

In the context of this study, we also sought to determine whether enrichment strategies could improve the sensitivity to change over time of these ADAS-Cog variants. Since the introduction of enrichment as part of inclusion criteria for early stages of AD in 2007 [21], enrichment biomarker strategies have been used in clinical trials to include subjects with prodromal AD (also called MCI due to AD). The enrichment strategy is aimed at detecting the presence of amyloidosis in the brain as a hallmark of AD [22, 23], for example by using CSF biomarkers [24] such as low Aβ and high Tau. As 60 % of subjects with MCI and 40 % of subjects with AD provided CSF, we used the available CSF baseline data to select enriched populations (i.e., subjects most resembling those to be recruited into clinical trials). This gave us the opportunity to also compare the performance of ADAS-Cog variants in enriched vs. non-enriched groups. For our analyses, t-Tau/Aβ ratio in the CSF was used as the primary enrichment strategy to determine populations with AD pathology (enriched). In addition, we looked at the impact of single-biomarker modalities such as Aβ alone, the marker of neurodegeneration Tau and the prominent genetic risk factor for sporadic AD, the *ApoE4* allele [25]. It should be noted that these are not defined as enrichment biomarkers in the research criteria for prodromal AD [16].

There is an increasing body of evidence showing that AD starts decades before the clinical symptoms become apparent [26]. With new putative therapies aimed at slowing disease progression by early intervention, there is an increasing interest in identifying subjects at early stages of AD [27]. Advances in the biomarker field allow us to identify subjects with AD pathology before the clinical diagnosis is made [28]. It was hypothesised that using enriched populations in clinical trials reduces variability and increases the magnitude of cognitive decline over time, thus reducing the sample size necessary to detect a drug effect in clinical trials [29].

We analysed cognitive decline on the four ADAS-Cog variants (3-, 5-, 11- and 13-item scales) in ADNI subjects with MCI or mild AD who provided a CSF sample at baseline. The results showed that, in the MCI population, there was a minimal decline over 24 months on all ADAS-Cog variants. The impact of enrichment was detectable but subtle. The largest decline was less than 3 points over 24 months on ADAS-Cog 13 in enriched MCI. This provides some support for use of enrichment as a tool to identify subjects who are more likely to demonstrate a cognitive decline, although, at least in the case of the MCI, the impact upon such changes measured by ADAS-Cog variants is minimal and of questionable clinical relevance to be of any practical use for clinical trials with pharmacological intervention aimed at slowing decline of disease progression.

As expected, decline in mild AD was more pronounced than in MCI but still modest. ADAS-Cog 11 scores of subjects with mild AD declined 3.5 points over 12 months in our analysis. This seems to correspond to the rate of 2.4 points over 6 months reported by Doraiswamy et al. [1]. It should be noted that the rate of decline could be influenced by many factors, such as education, background medication and potentially attrition. The fact that ADNI participants were highly educated, with, on average, 15–16 years of education, has been reported previously [12]. In addition, many ADNI participants, particularly those with mild AD, may be receiving symptomatic treatment [30]. Even though these factors would be of interest, attempting evaluation of all these factors runs the risk that the ultimate sample size would be too small to provide any meaningful results. The impact of enrichment could not be evaluated, as over 90 % of subjects with mild AD enrolled in ADNI studies had positive AD pathology at baseline.

These results were also reflected on the MMSE scale: no worsening in the MCI set and modest worsening in the mild AD set over time. Meta-analysis of MMSE change in patients with mild to moderate AD has previously shown an annual rate of decline of 3.3 points [31]. Similar rates have been reported by other groups [32]. The rate of decline seen in our analysis is more modest, probably due to the relatively earlier stage of disease than in previously published studies. It should be noted, however, that the researchers in the above-mentioned studies analysed data of non-treated patients, while many ADNI participants, especially those with mild AD, were on background medication [30].

The SNR reflects the ratio between mean change and variability, thus allowing direct comparison of the sensitivity of the different ADAS-Cog variants. High SNR values reflect increased sensitivity to detect a change over time, and thus the ADAS-Cog variant with the highest SNR value should theoretically lead to an optimal instrument.

In MCI, the SNRs for each ADAS-Cog variant and for each enrichment strategy were relatively similar but low. The numerically largest SNRs were seen for the ADAS-Cog 5 and *ApoE4* enrichment. However, as the 95 % confidence intervals around each of the SNRs were broad, the results did not support a meaningful difference among groups.

We confirmed that the ADAS-Cog instrument is not sensitive to detect change over time at pre-dementia stages of the disease such as MCI (as shown by small SNRs), and therefore the impact of enrichment strategies was subtle. The numerically largest SNR was seen for the enriched set according to *ApoE4* risk. *ApoE* genotype is a well-described genetic risk factor for AD associated with early deficits in episodic recall, higher rates of cognitive decline before the diagnosis of MCI or AD, and age-related memory decline earlier in life (for review, see [33]). The presence of an *ApoE4* allele doubles the risk of progression from normal cognition to onset of clinical symptoms [34]. In addition, the *ApoE4* allele has been associated with a faster cognitive deterioration in several, but not all, studies of patients with AD. Therefore, *ApoE4* genotype is often used as a stratification factor in clinical trials testing novel therapies and, interestingly, is being used as an enrichment strategy in the ongoing TOMMORROW trial [35]. However, as the 95 % confidence interval was broad, it can be concluded that the benefit of such enrichment in MCI seems negligible for this particular instrument. We do not believe that enrichment would allow for significant reduction of the required sample size in MCI if subjects were to be tested using ADAS-Cog.

The SNRs were larger in subjects with mild AD than in subjects with MCI. Similarly to MCI, the numerically highest SNRs were generally seen for the *ApoE4*-enriched set across all variants. Interestingly, the highest SNRs were seen for both the 5-item and 13-item ADAS-Cog variants. This was supported by the *p* values with the strongest evidence for a difference seen when comparing the 11- vs. 13-item variants and the 3- vs. 5-item variants. One could speculate that the difference between variants is driven more by the two additional items (Delayed Word Recall and Digit Cancellation) than by removing the items at ceiling in early stages of AD, suggesting the increase in magnitude of the change outweighs the increase in variability.

### Limitations of this analysis

In addition to the high education level of ADNI participants [12, 36] discussed earlier, it is also acknowledged that the participating sites in the ADNI study are experienced. This is reflected by the observation that there is less variability in the scores and a more reliable diagnosis (>90 % subjects with mild AD had positive AD pathology). This is contrary to recent phase III clinical trials with potential anti-amyloid therapies, which have shown that enrolling participants without evidence of amyloid deposition is common [37].

In mild AD, the number of subjects attending follow-up visits beyond the first year dropped significantly and did not provide a sufficient number of subjects for meaningful analyses. Therefore, changes over time beyond 12 months need to be interpreted with caution. Per regulatory requirement, a minimum of 18 months of treatment is required in clinical trials aimed at disease modification.

It was the purpose of this investigation to analyse subjects with follow-up data. The impact of the clinical characteristics of subjects who withdrew prematurely on change over time was not evaluated, as this would have required assumptions regarding the follow-up data. Such assumptions are the subject of an ongoing scientific debate.

We recognize that the value of the ADAS-Cog variants in our dataset is being judged solely on the basis of a longitudinal change over time, regardless of the treatment status of included subjects. Therefore, the outcome may not necessarily directly translate into a variant with the highest sensitivity to detect differences between treatments.

It must also be noted that the ADAS-Cog variants analysed in this study were collected in the context of ADAS-Cog 11, so the items are likely prone to specific order effects. Moreover, the 13- and 5-item variants contained additional two items that were tested outside the original ADAS-Cog 11. The order effect, where exposure to earlier tests influences performance on later tests, is a well-known phenomenon in cognitive testing [38].

## Conclusions

Whilst the cognitive domain expansion or reduction of the ADAS-Cog is in principle a worthwhile endeavour, in practice the use of these measures has been less successful. The outcome of our analyses showed that the possible utility of these novel ADAS-Cog variants is less than compelling, particularly in MCI. The impact of enrichment on cognitive decline in MCI using the latest research criteria [16] was subtle. In mild AD, adding items known to be impaired at early stages of the disease seems to provide more benefit than removing items on which subjects score close to ceiling.

## Additional files

Additional file 1:
**Items of ADAS-Cog variants.** Supplementary table providing details about items of ADAS-Cog variants. (DOCX 15 kb) Additional file 2:
**ADAS-Cog 11 scores at each visit and change from baseline for subjects with mild AD.** Supplementary table providing details of ADAS-Cog 11 scores at each visit and change from baseline for subjects with mild AD. (DOCX 15 kb) Additional file 3:
**MMSE score up to 24 months for subjects with MCI and mild AD.** Supplementary table providing details of MMSE score up to 24 months for subjects with MCI and mild AD. (DOCX 14 kb) Additional file 4:
***P*** **values for comparison of signal-to-noise ratios of ADAS-Cog 11 and ADAS-Cog 5 vs. other variants.** Supplementary table providing *p* values for comparison of signal-to-noise ratios of ADAS-Cog 11 and ADAS-Cog 5 vs. other variants. (DOCX 16 kb)

## Abbreviations

Aβ: amyloid-β
AD: Alzheimer’s disease
ADAS-Cog: Alzheimer’s Disease Assessment Scale–Cognitive subscale
ADNI: Alzheimer’s Disease Neuroimaging Initiative
*ApoE4*: apolipoprotein E ε4
CDR-SB: Clinical Dementia Rating Sum of Boxes
CFB: change from baseline
CI: confidence interval
CSF: cerebrospinal fluid
IWG: International Working Group
MCI: mild cognitive impairment
MMSE: Mini Mental State Examination
p-Tau: phosphorylated Tau
SD: standard deviation
SNR: signal-to-noise ratio
t-Tau: total Tau

## Footnotes

1 The text in italics in the Data source section is required by the ADNI consortium.
