# Supplementary material for: Alzheimer’s Disease Assessment Scale–Cognitive subscale variants in mild cognitive impairment and mild Alzheimer’s disease: change over time and the effect of enrichment strategies
Source: Alzheimers Res Ther. 2016 Feb 12;8:8. doi: 10.1186/s13195-016-0170-5 (PMC4751673; doi:10.1186/s13195-016-0170-5)
Supplement: Additional file 1: — Items of ADAS-Cog variants. Supplementary table providing details about items of ADAS-Cog variants. (DOCX 15 kb) [file 13195_2016_170_MOESM1_ESM.docx]

Additional file 1 Items of ADAS-cog variants

| Item Nr. | Item (score range) | 11-items | 3-items | 13-items | 5-items |
| --- | --- | --- | --- | --- | --- |
| 1 | Word Recall (0-10) | X | X | X | X |
| 2 | Commands (0-5) | X |  | X |  |
| 3 | Constructional Praxis (0-5) | X |  | X |  |
| 4 | Delayed Word Recall (0-10) |  |  | X | X |
| 5 | Object & Finger Naming (0-5) | X |  | X |  |
| 6 | Ideational Praxis (0-5) | X |  | X |  |
| 7 | Orientation (0-8) | X | X | X | X |
| 8 | Word Recognition (0-12) | X | X | X | X |
| 9 | Remembering Test Instructions (0-5) | X |  | X |  |
| 10 | Spoken language ability (0-5) | X |  | X |  |
| 11 | Word-finding difficulty (0-5) | X |  | X |  |
| 12 | Comprehension (0-5) | X |  | X |  |
| 14 | Digit Cancellation (0-5) |  |  | X | X |
|  | **Total score achievable** | **70** | **30** | **85** | **45** |

Note: item number based on order in ADNI

ADAS-cog, Alzheimer’s Disease Assessment Scale–cognitive subscale; ADNI, Alzheimer’s Disease Neuroimaging Initiative.
