# Supplementary material for: Alzheimer’s Disease Assessment Scale–Cognitive subscale variants in mild cognitive impairment and mild Alzheimer’s disease: change over time and the effect of enrichment strategies
Source: Alzheimers Res Ther. 2016 Feb 12;8:8. doi: 10.1186/s13195-016-0170-5 (PMC4751673; doi:10.1186/s13195-016-0170-5)
Supplement: Additional file 2: — ADAS-Cog 11 scores at each visit and change from baseline for subjects with mild AD. Supplementary table providing details of ADAS-Cog 11 scores at each visit and change from baseline for subjects with mild AD. (DOCX 15 kb) [file 13195_2016_170_MOESM2_ESM.docx]

**Additional file 2 ADAS-cog11 scores at each visit and change from baseline for mild AD subjects***

|  | **Mild AD**  **(‘non-enriched’)** | | | **Mild AD+**  **(‘enriched’)** | | |
| --- | --- | --- | --- | --- | --- | --- |
|  | **N** | **ADAS-cog11** | **CFB** | **N** | **ADAS-cog11** | **CFB** |
| Baseline/ Screen | 97 | 19.66 ± 6.30 |  | 90 | 20.14 ± 6.26 |  |
| Mo 6 | 95 | 21.19 ± 6.98 | 1.7 ± 4.37 | 88 | 21.61 ± 7.01 | 1.6 ± 4.43 |
| Mo 12 | 97 | 23.19 ± 8.91 | 3.5 ± 5.59 | 90 | 23.86 ± 8.86 | 3.7 ± 5.73 |
| Mo 24 | 40 | 26.13 ± 11.97 | 8.3 ± 8.96 | 37 | 26.93 ± 12.07 | 8.7 ± 9.13 |

*Data are presented as mean ± SD.

Increased score on ADAS-cog11 (maximum total score 70) indicates cognitive worsening.

Change from baseline was calculated as a mean of all individual changes from baseline.

AD, Alzheimer’s disease; ADAS-cog, Alzheimer’s Disease Assessment Scale–cognitive subscale; CFB, change from baseline; Mo, month; SD, standard deviation.
