# Supplementary material for: Alzheimer’s Disease Assessment Scale–Cognitive subscale variants in mild cognitive impairment and mild Alzheimer’s disease: change over time and the effect of enrichment strategies
Source: Alzheimers Res Ther. 2016 Feb 12;8:8. doi: 10.1186/s13195-016-0170-5 (PMC4751673; doi:10.1186/s13195-016-0170-5)
Supplement: Additional file 3: — MMSE score up to 24 months for subjects with MCI and mild AD. Supplementary table providing details of MMSE score up to 24 months for subjects with MCI and mild AD. (DOCX 14 kb) [file 13195_2016_170_MOESM3_ESM.docx]

**Additional file 3 MMSE score up to 24 months for MCI and mild AD subjects***

|  | **MCI** | **MCI+** | **MCI-** | **Mild AD** | **Mild AD+** |
| --- | --- | --- | --- | --- | --- |
| Baseline/ Screen | 27.85 ± 1.75 | 27.41 ± 1.82 | 28.37 ± 1.52 | 23.19 ± 1.99 | 23.14 ± 1.99 |
| Mo 6 | 27.48 ± 2.10 | 26.82 ± 2.33 | 28.26 ± 1.46 | 22.43 ± 3.15 | 22.27 ± 3.11 |
| Mo 12 | 27.46 ± 2.27 | 26.71 ± 2.43 | 28.34 ± 1.69 | 20.71 ± 4.74 | 20.39 ± 4.72 |
| Mo 24 | 26.91 ± 2.90 | 26.00 ± 3.21 | 27.97 ± 2.05 | 18.68 ± 5.27 | 18.46 ± 5.37 |

*Data are presented as mean ± SD.

Decreased MMSE score (maximum total score 30) indicates cognitive worsening.

AD, Alzheimer’s disease; MCI, mild cognitive impairment; MMSE, mini-mental state examination; Mo, month; SD, standard deviation.
