# Supplementary material for: Alzheimer’s Disease Assessment Scale–Cognitive subscale variants in mild cognitive impairment and mild Alzheimer’s disease: change over time and the effect of enrichment strategies
Source: Alzheimers Res Ther. 2016 Feb 12;8:8. doi: 10.1186/s13195-016-0170-5 (PMC4751673; doi:10.1186/s13195-016-0170-5)
Supplement: Additional file 4: — P values for comparison of signal-to-noise ratios of ADAS-Cog 11 and ADAS-Cog 5 vs. other variants. Supplementary table providing p values for comparison of signal-to-noise ratios of ADAS-Cog 11 and ADAS-Cog 5 vs. other variants. (DOCX 16 kb) [file 13195_2016_170_MOESM4_ESM.docx]

**Additional file 4 *P*-values for comparison of signal-to-noise ratios* of ADAS-cog11 and ADAS-cog5 vs other variants**

Detailed legend: *P*-Values for comparison of signal-to-noise ratios* of ADAS-cog11 vs other variants and of ADAS-cog5 vs other variants

|  | **MCI (24 mo)** | | | | |
| --- | --- | --- | --- | --- | --- |
|  | **Comparison with ADAS-cog11** | | | **Comparison with ADAS-cog5** | |
| **Variants:** | **11 vs 3** | **11 vs 5** | **11 vs 13** | **5 vs 3** | **5 vs 13** |
| Non-enriched | 0.26 | 0.45 | 0.65 | 0.99 | 0.40 |
| t-tau/Abeta > 0.39 | 0.47 | 0.47 | 0.54 | 0.78 | 0.57 |
| Abeta < 192 | 0.31 | 0.42 | 0.57 | 0.92 | 0.45 |
| ApoE4 + | 0.90 | 0.30 | 0.18 | 0.24 | 0.83 |
| t-tau > 93 | 0.34 | 0.15 | 0.20 | 0.30 | 0.26 |
| P-tau > 23 | 0.26 | 0.48 | 0.74 | 0.91 | 0.38 |
|  | **Mild AD (12 mo)** | | | | |
| **Variants:** | **11 vs 3** | **11 vs 5** | **11 vs 13** | **5 vs 3** | **5 vs 13** |
| Non-enriched | 0.63 | 0.65 | 0.11 | 0.12 | 0.70 |
| t-tau/Abeta > 0.39 | 0.81 | 0.54 | 0.11 | 0.13 | 0.88 |
| Abeta < 192 | 0.90 | 0.47 | 0.10 | 0.14 | 0.96 |
| ApoE4 + | 0.17 | 0.79 | 0.20 | 0.15 | 0.27 |
| t-tau > 93 | 0.78 | 0.10 | 0.02 | 0.01 | 0.51 |
| P-tau > 23 | 0.67 | 0.58 | 0.08 | 0.09 | 0.78 |

*Corrected for covariates: baseline ADAS-cog and MMSE scores, age, gender, and ApoE4 risk category.

Abeta, amyloid beta; AD, Alzheimer’s disease; ADAS-cog, Alzheimer’s Disease Assessment Scale–cognitive subscale; MCI, mild cognitive impairment; MMSE, mini-mental state examination; Mo, month.
